# Central retinal volume derived from optical coherence tomography as a potential predictor of mortality in the old-aged population– results from the German AugUR study

**DOI:** 10.1007/s00417-025-06924-4

**Published:** 2025-08-05

**Authors:** Klaus J. Stark, Martina E. Zimmermann, Horst Helbig, Iris M. Heid, Caroline Brandl

**Affiliations:** 1 Department of Genetic Epidemiology, University of Regensburg, Franz-Josef-Strauß-Allee 11, 93053 Regensburg, Germany; 2 Department of Ophthalmology, University Hospital Regensburg, Regensburg, Germany

**Keywords:** Cohort study, Old-aged population, AugUR, Mortality, OCT, Central retinal volume, Risk factors

## Abstract

**Purpose:**

To estimate mortality risk depending on central retinal volume (CRV) from optical coherence tomography (OCT) in a German cohort of the old-aged population.

**Methods:**

In the AugUR study, a prospective population-based cohort study in individuals aged 70–95 years at baseline, we conducted multimodal retinal imaging, including spectral-domain OCT. Heidelberg Spectralis-derived CRV measurements from first examinations of 2,166 participants were included in the analyses. Within the observation period (median 5.9-years), 374 participants died. Association between CRV at baseline and mortality was analysed with Kaplan-Meier curves and Cox proportional hazard regression.

**Results:**

Decrease in CRV was associated with increased all-cause mortality risk. In a full model with age, sex, body weight, body size, OCT scan focus, age-related macular degeneration, smoking, cardiovascular disease, diabetes, and hypertension, hazard ratio per standard deviation lower CRV was 1.17. Cardiovascular death was not associated with CRV in the full model. However, other causes for death except cardiovascular reasons showed association with lower CRV (hazard ratio 1.25). In addition, the association was significant in those who had already exceeded their expected life expectancy (hazard ratio 1.21) but not in women below 83 years and men below 78 years, respectively.

**Conclusion:**

This study indicates that lower CRV, which can be easily and automatically derived from OCT images, is a potential predictor for mortality in the old-aged population. This effect occurs independently of cardiovascular disease.

**Supplementary Information:**

The online version contains supplementary material available at 10.1007/s00417-025-06924-4.

## Key Messages

*****What is known***:**

- Retinal layer thicknesses derived from optical coherence tomography scans were reported to be associated with mortality in the UK Biobank Study

*****What is new***:**

- Lower central retinal volume is associated with increased mortality risk in an old-aged population
- This finding is independent of other competing risk factors, particularly cardiovascular disease

**Supplementary Information:**

The online version contains supplementary material available at 10.1007/s00417-025-06924-4.

## Introduction

Optical coherence tomography (OCT) is a non-invasive imaging technique to visualize the human retina and to derive features of retinal compositions [1]. It not only allows detailed qualitative assessment of disease-associated features but also quantitative assessment of retinal layer thicknesses and retinal volume with quality control measurements [2]. The human retina is influenced by environmental and genetic factors, which can manifest in various diseases, e.g. retinal degenerative diseases [3]. Therefore, it is important to differentiate between physiological and pathological changes in the retina.

Age itself is one driver of changes in retinal compositions, with thickening in the fovea and thinning of the inner and outer macula [4–6]. Non-pathological changes in retinal thickness could provide insight into biological aging which is often different from chronological age [7]. The retina was in focus of research dealing with biological aging for the last years, especially applying modern machine learning and deep neural network algorithms [8–11]. Recent studies showed that biological aging markers derived from retinal images are predictors of morbidity and mortality [11, 12]. Zekavat et al. reported associations between retinal layers derived from Spectral Domain Topcon 3D OCT with incident mortality in the UK Biobank including data from 44,823 individuals with a mean age of 56.8 years and a median 10-year mortality follow-up period (1,746 deceased) [12].

While previous studies have identified associations between retinal layer thicknesses and mortality, the role of overall central retinal volume (CRV), particularly in older populations, remains underexplored. Here, we aimed to analyse the association between CRV and all-cause as well as cardiovascular and non-cardiovascular mortality in 2,166 participants of the German AugUR study with individuals aged 70 years and older [13].

## Materials and methods

### AugUR cohort study description

The German AugUR study (*A*ltersbezogene *U*ntersuchungen zur *G*esundheit der *U*niversität *R*egensburg) is a prospective study of the general old-aged population in and around the city of Regensburg, Bavaria. AugUR focuses on chronic diseases and associated risk factors in the population aged 70 to 95 years at baseline. Details on the study were published earlier [13–17]. In brief, 1,133 participants were included in the AugUR1 survey between 2013 and 2015, and 1,316 in the independent second survey, AugUR2, between 2017 and 2019. For AugUR1, three year follow-up was conducted between 2016 and 2018 with 733 participants.

The AugUR study was approved by the Ethics Committee of the University of Regensburg, Germany (vote 12-101-0258). The study complies with the 1964 Helsinki declaration and its later amendments. All participants provided informed written consent.

### Analysis sample

**Fig. 1:**
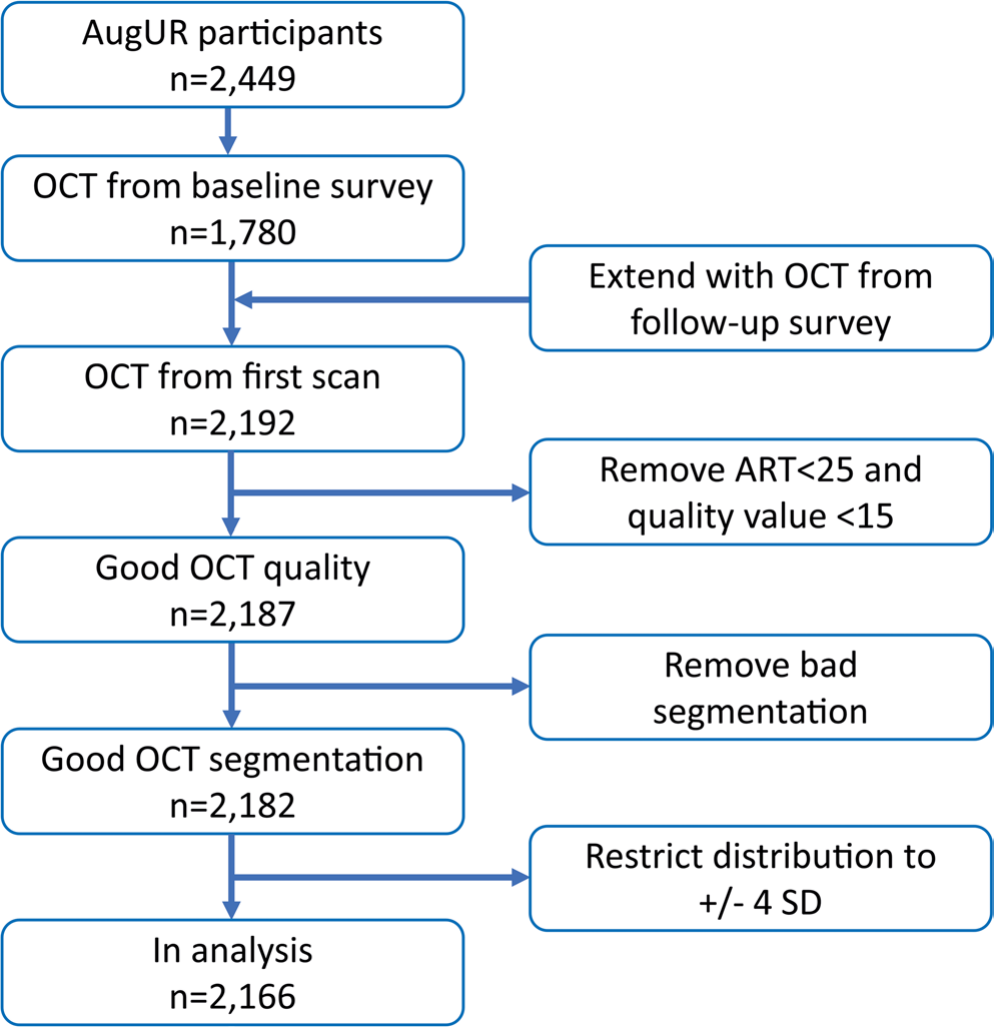
Flow chart of AugUR recruitment and generating OCT analysis data set for the right eye (*n* = 2,166)

AugUR recruitment started in 2013 with spectral-domain OCT examinations available since 2014 with a total number of 1,780 participants with OCT scans of the right eye at baseline. To maximize sample size, we included all first OCT recordings per participant from baseline and the follow-up examination resulting in 2,192 participants with OCT data (extended baseline). After quality control steps, 2,166 participants with high-quality OCT scans of the right eye remained (Fig. 1).

### AugUR study program

Macular cube volumetric SD-OCT scans with 49 Raster lines at 20 × 20° (about 6 × 6 mm) with an interscan distance of 120 μm between the 49 B-scans with 30 automated real-time (ART) repetitions centred on the fovea, were acquired after mild mydriasis via OCT Spectralis platform (Heidelberg Engineering, Heidelberg, Germany) and imported into the Heidelberg Eye Explorer software, version 1.10.4.0 (Heidelberg Engineering). The built-in feature for automated volume derivation was applied. Central retinal volume (CRV) was calculated between Bruch’s membrane (BM) and inner limiting membrane (ILM) summed for all nine Early Treatment Diabetic Retinopathy Study (ETDRS) segments, spanning a zone with about 6-mm diameter around the fovea [18, 19] (Supplementary Fig. 1). Volumes from combined ETDRS segments for seven distinct layers of the central retina were directly exported from the Eye Explorer software without manual curation of the boundaries [15] (NFL, nerve fibre layer; GCL, ganglion cell layer; IPL, inner plexiform layer; INL, inner nuclear layer; OPL, outer plexiform layer; ONL, outer nuclear layer; RPE, retinal pigment epithelium). The quality control steps (Fig. 1 and Supplementary Fig. 2A-D) for OCT-derived volume data consisted of (i) exclusion of all OCT scans with ART metrics < 25 and internal quality value < 15 [2], (ii) removal of all data with bad segmentation defined by “0” in one of the retinal layer thickness measurements, and (iii) restriction to volume measurements +/- 4 SD to remove outliers (Supplementary Fig. 2C) resulting in an approximately normal distribution (Supplementary Fig. 2D). For reasons of comparability and interpretability, CRV values were divided by its negative standard deviation (SD) to obtain hazard ratio (HR) estimates > 1 per SD lower CRV. Scan focus on the macula was extracted from OCT data and was used as a proxy variable for refractive error (given in dioptres, D).

Colour fundus photography of the central retina and assessment of features of age-related macular degeneration (AMD) were conducted as described previously [14]. AMD status per eye was classified according to the Three Continent AMD Consortium Severity Scale (3CACSS) as no AMD, mild/moderate/severe early AMD, or late AMD (geographic atrophy or neovascular AMD) [20].

General medical examinations at the study centre included height, weight and blood pressure. A questionnaire conducted as in-person interview included information on general chronic diseases, medication intake and lifestyle factors like current and former smoking. Medical conditions were recorded via participants’ self-report using the question ‘Has a physician ever diagnosed one of the following diseases?’. Coronary artery disease (CAD) was defined if at least one of the following conditions were reported by the participants: myocardial infarction, percutaneous coronary intervention, or coronary artery bypass surgery. Cardiovascular disease (CVD) was defined as CAD or stroke. Hypertension was defined as blood pressure ≥ 140/90 mmHg or if the individual reported a prior hypertension diagnosis and antihypertensive medication intake [21]. Individuals with self-reported diabetes and/or antidiabetic medication intake were defined as having diabetes [22].

### Mortality survey and causes of death in the AugUR study

For all AugUR study participants central registers were queried in November/December 2023 to determine individual mortality. Observation time was censored for survivors from AugUR1 on November 15th, 2023, and to those from AugUR2 on December 31 st, 2023, resulting in a follow-up period of up to 10.6 years. Based on death certificates, causes of death were classified as ‘cardiovascular’ if they included International Statistical Classification of Diseases, 10th Revision codes I00-I99. Since current life expectancy in Germany is 83 for women and 78 years for men, respectively, (https://www.destatis.de/, queried 2025-01-08) we additionally analysed risk for premature mortality and death in old age based on these sex-specific age cut-offs.

### Statistical analysis

Data management and statistical analyses were performed using SAS 9.4 software (SAS Institute Inc., Cary, NC, USA), IBM SPSS Statistics for Windows, Version 29.0.1.1 (IBM Corp., Armonk, NY, USA) and R statistical software v.4.5.0 (R Core Team 2025). For correlation analyses, Persons’ coefficient r was calculated. Kaplan-Meier curves were generated by dividing the study cohort at the median of the CRV distribution. Survival curves with 95% confidence intervals (95% CI) were generated with the R packages survival v3.8-3 and ggplot2 v3.5.2. Log-rank test was applied for the differences of the two survival curves. Cox proportional hazard survival regression analyses were performed to model the time to event data for per SD decrease in central retinal volume. AMD and smoking status were included in the models as categorical variables. 95% CI for HR estimates were calculated as $\:{e}^{\left(b\pm\:1.96*SE\right)}$. For testing of differences between two HRs the Wald test $\:Z=\frac{\text{ln}\left(HR1\right)-\text{l}\text{n}\left(HR2\right)}{\sqrt{{SE1}^{2}+{SE2}^{2}}}$ was applied und one-sided p_Diff_ was reported. Competing risk regression according to Fine and Gray [23] was performed using R package cmprsk in version 2.2–12.

## Results

### Characteristics of individuals in the AugUR study population

From the 2,166 individuals with high-quality OCT data on the right eye available (52.9% women), 374 deceased during a median follow-up time of 5.9 years (interquartile range, 4.6–6.9 years; maximum 10.6 years). CVD mortality was recorded for 107 individuals (28.6% of death causes). The characteristics of all AugUR participants at baseline and for mortality data are shown in Table 1. Decrease in CRV was only weakly correlated with age in our study data (*r* = 0.12; Supplementary Fig. 3). Among those study participants who did not die during the observation period compared to those who did die for various reasons showed slight differences in CRV distribution (Supplementary Fig. 4). Since AMD forms are known to influence macular thickness, distribution of CRV in different AMD stages was analysed. Late AMD, especially geographic atrophy led to lower CRV (Supplementary Fig. 5).

**Table 1:** Characteristics of AugUR participants with high-quality OCT scans of the right eye

Characteristic	Overall (*n*=2,166)	Missing values
General descriptives		
Age, years [min– max]	78.8 ± 4.9 [70.3– 98.3]	0
Sex, men	1,020 (47.1%)	0
Body height, cm	165.1 ± 8.9	13
Body weight, kg	75.6 ± 14.2	14
Never smoked	1,190 (55.2%)	12
Former smokers	847 (39.3%)	12
Current smokers	117 (5.4%)	12
OCT parameters		
CRV, mm³ [min– max]	8.40 ± 0.55 [5.98– 10.86]	0
CRV, per negative SD [min– max]	-15.28 ± 1.00 [-19.76– -10.88]	0
Scan Focus [D]	0.20 ± 2.03	0
Image quality	28.65 ± 3.37	0
ART	29.91 ± 2.43	0
Diseases		
Early AMD / Late AMD	390 (19.5%) / 99 (5.0%)	166
CAD	325 (15.1%)	16
CVD	468 (21.8%)	16
Diabetes mellitus	461 (21.3%)	1
Hypertension	1,586 (73.3%)	3
Mortality data		
Median follow-up time [IQR]	5.9 [4.6;6.9]	0
All-cause death	374 (17.3%)	0
CVD death	107 (4.9%)	267
Non-CVD death	241 (11.1%)	133
Premature death	77 (3.6%)	297
Death in old age	297 (13.7%)	77

Continuous values are means ± standard deviation if not noted otherwise; categorial variables are total numbers and percent in brackets. Missing values per variable are shown in the right column.

*OCT* optical coherence tomography; *CRV* central retinal volume; *SD* standard deviation; *D* dioptres from the OCT scan focus as a proxy for refractive error; *ART* automatic real time metrics; *AMD* age-related macular degeneration; *CAD* coronary artery disease; *CVD* cardiovascular disease (CAD and/or stroke); *IQR* interquartile range [25^th^ and 75^th^ percentile]. For mortality subcategories, unknown reasons for death were set as missing. In addition, for CVD mortality all other reasons were set as missing, whereas for non-CVD mortality CVD death was set as missing. Premature death was defined as below the age of 83 for women and 78 years for men, respectively. Conversely, death in old age was defined above these age cut-offs

**Fig. 2:**
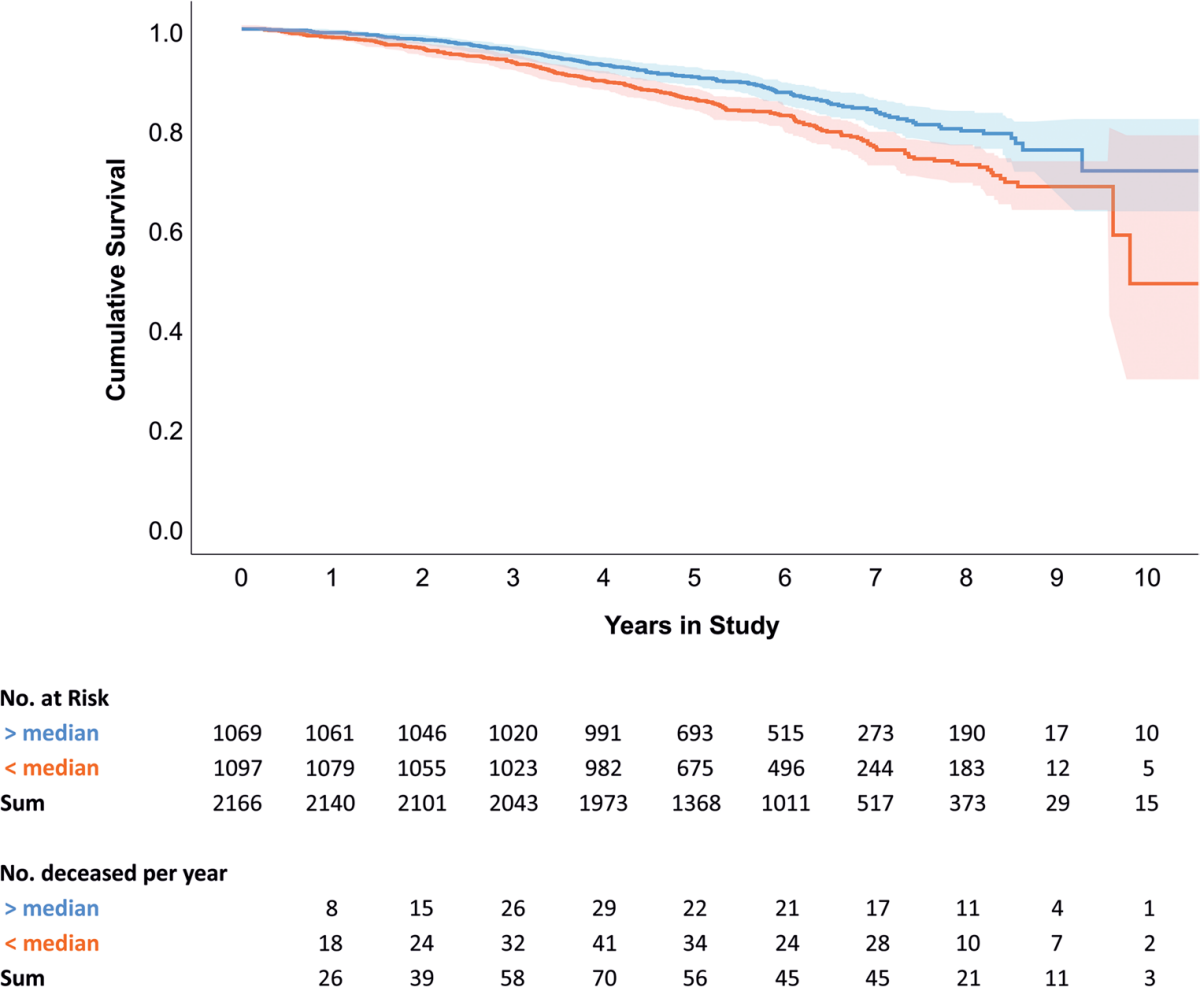
Kaplan-Meier estimates of cumulative survival in persons categorized by median of central retinal volume. Survival functions (lines) and 95% CI (shades) are depicted. From the initial 2,166 persons, 374 died within 10 years. Numbers of persons at risk and all-cause deaths per year in higher (blue) and lower (red) than the median central retinal volume are shown. Log rank test: *p* = 3.26*10^−4^

**Fig. 3:**
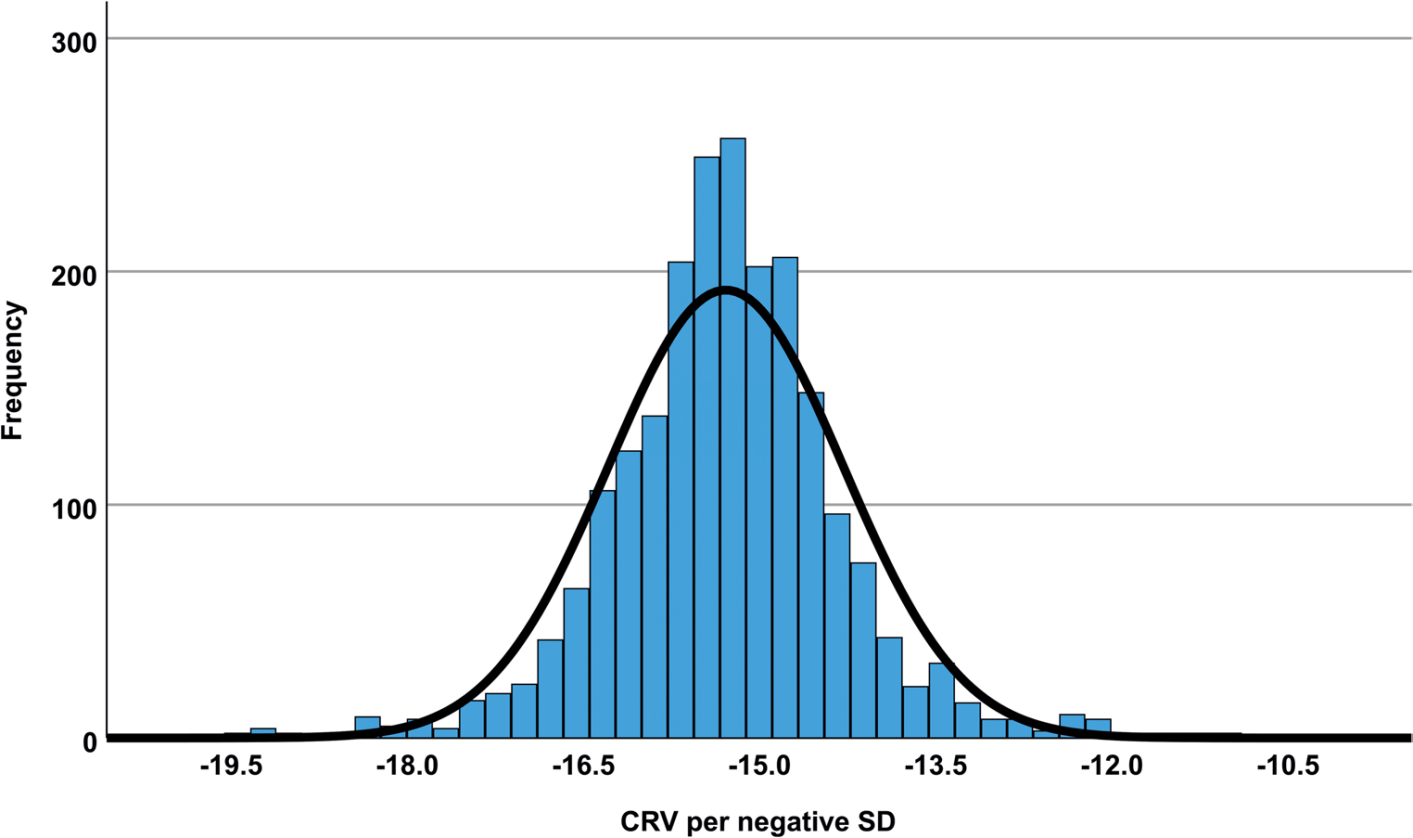
Distribution of central retinal volume (CRV) of the right eye (*n* = 2,166) after quality control steps and normalisation on negative standard deviation (SD) as used in analyses. Normal distribution derived from mean ± SD is depicted as a curve

Participants with a CRV below the median value of 8.40 mm³ had a significantly higher proportion of mortality compared to participants with a higher CRV (log-rank p-value = 3.26*10^−4^; Fig. 2). For Cox proportional hazard survival regression analyses we used the per negative SD normalised CRV, ranging 8.88 mm³ from minimum to maximum (Fig. 3). This allows to quantify the mortality risk per SD decreased CRV. Different models were applied to test for potential confounding factors and competitive risk (Table 2):

**Table 2:** Risk of mortality associated with central retinal volume

Model	All-cause	CVD	Non-CVD	Premature	Old age
Unadjusted	**1.17 [1.06; 1.30]** *p* = 0.002 (*n* = 374) *	**1.25 [1.04; 1.51]** *p* = 0.019 (*n* = 107) *	**1.18 [1.04; 1.34]** *p* = 0.011 (*n* = 241) *	0.97 [0.77; 1.22] *p* = 0.795 (*n* = 77)	**1.24 [1.11; 1.39]** *p* = 0.00014 (*n* = 297) *
Model 1	**1.14 [1.03; 1.26]** *p* = 0.012 (*n* = 374) *	1.21 [1.00; 1.46] *p* = 0.051 (*n* = 107)	**1.15 [1.02; 1.31]** *p* = 0.028 (*n* = 241) *	1.05 [0.83; 1.33] *p* = 0.691 (*n* = 77)	**1.19 [1.06; 1.33]** *p* = 0.003 (*n* = 297) *
Model 2	**1.19 [1.06; 1.32]** *p* = 0.002 (*n* = 325) *	1.17 [0.96; 1.43] *p* = 0.132 (*n* = 92)	**1.27 [1.10; 1.46]** *p* = 0.001 (*n* = 210) *	1.13 [0.88; 1.46] *p* = 0.340 (*n* = 70)	**1.22 [1.08; 1.38]** *p* = 0.002 (*n* = 255) *
Model 3	**1.17 [1.05; 1.30]** *p* = 0.006 (*n* = 321) *	1.15 [0.94; 1.39] *p* = 0.168 (*n* = 89)	**1.25 [1.09; 1.43]** *p* = 0.002 (*n* = 209) *	1.13 [0.87; 1.46] *p* = 0.385 (*n* = 68)	**1.21 [1.07; 1.36]** *p* = 0.003 (*n* = 253) *

Shown are hazard ratios (HR) and 95% confidence intervals (CI) in square brackets as well as p-values from Cox regression models and number (n) of deaths. Unadjusted, only central retinal volume (per standard deviation decrease); model 1, adjusted for age and sex; model 2, additionally adjusted for body weight, body size, scan focus, age-related macular degeneration; model 3, additionally adjusted for smoking, CVD, diabetes, hypertension. CVD, cardiovascular disease. Significant findings (*p* < 0.05) are marked with * and corresponding HR estimates with its 95% CI in bold font

(i) unadjusted,
(ii) age and sex (model 1),
(iii) additional factors with a potential influence on the central retinal volume (body weight, body size, scan focus, AMD; model 2), and.
(iv) competitive risk factors (smoking, CVD, diabetes, hypertension; model 3).

Each SD decrease in CRV increased all-cause mortality risk by 17% (*p* = 0.002). This finding was stable in the different models. In the full model 3, significant associations between CRV and all-cause mortality was found (HR = 1.17, 95% CI = 1.05–1.30, *p* = 0.006). CVD mortality association with CRV was significant only in the unadjusted analysis (HR = 1.25, 95% CI = 1.04–1.51, *p* = 0.019). In contrast, association between CRV and non-CVD mortality was significant in all three models (full model 3: HR = 1.25, 95% CI = 1.09–1.43, *p* = 0.002). However, HR estimates between CVD and non-CVD mortality were not significantly different (full model 3: *p* = 0.37). Competing risk analysis, considering CVD mortality as the competing risk, revealed only a slight attenuation of the effect on non-CVD mortality (full model 3: HR = 1.22, 95% CI = 1.06–1.42, *p* = 0.007). Old age mortality was associated with CRV (full model 3: HR = 1.21, 95%CI = 1.07–1.36, *p* = 0.003), but not premature death (full model 3: HR = 1.13, 95%CI = 0.87–1.46. *p* = 0.385). HR estimates between premature and old age mortality were not significantly different (full model 3: *p* = 0.38). It should be noted that the number of events decreased as variables with missing values (Table 1) were added to the models and, therefore, power was limited to reach nominal significance in these models between different death reasons (Table 2, number of events in brackets).

Since CRV is a quantitative OCT-derived parameter reflecting the thickness of the whole central retina, we analysed correlations with volumes of seven distinct retinal layers around the macula. Highest correlation could be found with IPL (*r* = 0.82). Other inner retinal layers also showed correlation with CRV *r* > 0.6, namely NFL, GCL, and INL (Supplementary Fig. 6). Correlation with outer retinal layer volumes were modest or weak (OPL, ONL, RPE).

## Discussion

Our study highlights OCT-based measurement of the CRV as a marker for mortality risk in an old-aged population. Kaplan-Meier analysis showed a significant increase in mortality risk for those with CRV values below the median of the AugUR study participants’ distribution. In Cox proportional hazard survival regression analyses significant associations between decrease in CRV and higher mortality risk were found. After full adjustment for confounding factors (smoking, CVD, diabetes, hypertension) and variables potentially influencing the retinal composition (body weight, body size, scan focus, AMD), we found strong association between lower CRV and increased non-CVD mortality. However, no association between CRV and CVD mortality in adjusted models was observed, and CVD mortality was no competing risk factor for non-CVD mortality. It has to be mentioned that the association between CRV and non-CVD mortality led to higher HR estimates in our model 2 with addition of body height, body weight, scan focus and AMD (HR = 1.27) compared to the age and sex-adjusted model 1 (HR 1.15). Additional adjusting for cardiovascular risk factors (model 3) did not substantially attenuate the association between lower CRV and higher non-CVD mortality risk (HR = 1.25; Table 2). This points to a mechanism beyond CVD for the association of lower CRV with increased mortality risk in our study sample.

In addition, premature mortality was not predicted by CRV, whereas in contrast mortality risk in participants older than the expected sex-specific life expectancy was associated with lower CRV (Table 2). In the case of premature versus old age mortality one has to consider that our study sample is aged 70 years or older at baseline. Therefore, premature death before the age of 70 is not covered in our study. Furthermore, ‘premature’ and ‘old-age mortality’, as defined here, are only surrogate parameters for unknown underlying mechanisms. Other age-dependent factors e.g. for frailty or disability should be considered in future analyses.

Recently, Zekavat et al. identified higher mortality risk for thinning of six calculated retinal thicknesses in the UK Biobank [12]. We limited our analysis to the total volume of the central retina rather than individual retinal layer thicknesses. This avoids a manual correction step of layer segmentation errors, as previously presented [15]. Additionally, by analysing only one feature instead of all retinal layers in nine ETDRS subfields, the problem of multiple testing in the statistics is avoided. Furthermore, measurement errors become more significant for smaller analysed segments.

We applied simple quality control steps on retinal layer composition by removal of measurements with retinal layers showing a value of “0” for thickness pinpointing an obvious error in the software’s automated segmentation. Together with the internal quality metrices of the Heidelberg Spectralis OCT (ART and quality value) and with the restriction of the mean CRV distribution +/- 4 SD (Supplementary Fig. 2C) led to high quality data with a slight loss of analysed eyes (1.2%).

Although CRV is not frequently measured and used in clinical practice, there are several reports in the literature that emphasise the usefulness of this marker. Wherever disturbances in the retinal composition occur, e.g. due to fluid deposits, the measurement of the total volume has advantages, as shown in the context of disease activity in neovascular AMD: change of macular retinal volume had shown high sensitivity and specificity to depict disease activity without the need for further characterization of intraretinal fluid [24]. For example, Gadde and colleagues reported threshold values for total retinal volume in the ETDRS map based on OCT measurements to detect macular oedema and showed that total retinal volume was highly correlated with oedema volume, making it usable in predictive models [25].

Several diseases and conditions affect different retinal layer thicknesses, e.g. at the outer retinal layers: early AMD was associated with thicker RPE/Bruch’s membrane complex, but also with thinner ONL and photoreceptor inner/outer segments + interdigitation zone in our AugUR study [15]. Also boundaries of layers were reported to be affected by early AMD stages, e.g. the ILM [26], and the curvature of the RPE was shown to be affected in early and intermediate AMD [27]. Late AMD forms lead e.g. to the loss of cell layers in geographic atrophy resulting in lower CRV (Supplementary Fig. 5). Therefore, AMD stages must be considered to avoid distortions of CRV in the analyses, as we did in our Cox regression models. However, the differences in HR for mortality with and without AMD in the model was modest, despite the prevalence of all AMD forms with about 25%. It is worth to mention that total retinal volume did not show correlations with pre-clinical AMD in a previous study [28]. Here, distinct retinal layer thicknesses have an advantage to detect early forms of AMD as shown in our own study [15].

Physiological aging leads to changes in the human organism [29] and specifically in the retina [30]. It is known that age contributes to the macular volume decrease in older persons without retinal pathologies [6]. However, in the here presented study with participants aged 70 and older, the effect of age on CRV is only marginal and age did not substantially influence the association between CRV and mortality. Retinal aging and its potential to predict mortality was in the focus of recent research. Especially, differences between chronological age and age derived from retinal photographs called ‘retinal age gap’ is a promising biomarker. Recently, Zhu and colleagues showed in deep learning model data from UK Biobank fundus images that the retinal age gap was associated with increased all-cause and non-CVD mortality risk but not with CVD mortality [9], comparable to our findings for CRV. Another study in UK Biobank used OCT-derived deep neural networks and found strong association between the OCT age gap and mortality and claimed better performance of OCT-based models compared to models from fundus images [10]. One problem with these deep learning methods is the question of which features contribute to the model (‘black box) [31].

CRV covers the volume of the whole retina from ILM to BM in the macular-centred ETDRS grid. In our analyses, CRV is most strongly correlated with the IPL (*r* = 0.82) among all seven retinal layer volumes derived from the Eye Explorer software (Supplementary Fig. 6). Also, correlations *r* > 0.6 were observed with NFL, GCL and INL, but not with OPL, ONL and RPE. This points to a larger effect of the inner retinal layers on the variance of CRV in our study. For example, in recent other studies central nervous system diseases like multiple sclerosis showed associations with thinner inner retinal layers not only at the optical disc but also at the central retina [32, 33]. In other disease entities like major depressive disorder significant negative correlations between depressive symptoms and macular total retinal volume as well as NFL, GCL and IPL were shown [34]. Alzheimer’s disease is known to show associations with many OCT-derived measurements, including macular volume in advanced forms of the disease [35]. Therefore, CRV could be also a marker for neurological conditions, as the eye is an extension of the brain, reflecting both neurodegeneration and vascular implications that are detectable in the macula, including nerve fibre layer on the outside and the choroid on the inner side. In another study, data from Heidelberg Spectralis SD-OCT (together with layers separately) were analysed in the context of retinal neurodegeneration in patients with end-stage kidney disease. Here, retinal differences between haemodialysis patients and healthy controls were tested and total retinal volume showed borderline association with thinner values in patients and single layer volumes were nominally associated in their study, pointing to neurodegeneration in ganglion cells and nerve fibre layer that was also reflected in total retinal volume [36]. Weerts and colleagues analysed total retinal volume and other retinal markers in the context of heart failure with preserved ejection fraction (HFpEF) and found association between lower total retinal volume and HFpEF [37]. The authors hypothesized that total retinal volume “may relate to disease severity in HFpEF and may imply systemic consequences of the syndrome” [37]. Another example for systemic conditions associated with lower retinal layer thicknesses especially in the outer macular subfields is hypertension [38].

Together with the observation that CVD and its risk factors did not substantially attenuate the association of all-cause, non-CVD and late mortality, our results indicate that CRV is a marker for mortality risk beyond the cardiovascular spectrum in our old-aged cohort. If validated, CRV could serve as a simple and automated biomarker to assess mortality risk in the aging population and support risk stratification through non-invasive OCT screening in geriatrics.

### Strengths and limitations

We need to acknowledge limitations of the current study. First, the AugUR population does not represent the general population aged 70+, since there is a selection for the mobile and, therefore, healthier elderly because they had to come to the study centre and were able to actively participate in the study program for more than two hours. Therefore, the here presented results may be not generalizable to the real-life old-aged population with a higher degree of comorbidity and age-related degenerative processes.

Second, we only analysed the association of the CRV and not the single retinal layers with mortality. This limitation was deliberately accepted in order to establish a parameter that is easy to measure, without the need for curation of layer integrity and avoids multiple testing that occurs when seven separate layers in nine ETDRS subfield were analysed. In addition, CRV sums up all layer thicknesses potentially influenced by different disease entities.

Third, our analysis was restricted to the right eye with more OCT scans available and we cannot rule out that CRV differences between right and left eyes are present. Together with the fact that we used only one device (Heidelberg Spectralis) and differences between OCT hardware and software also could occur [39], replications of our findings in other studies using the Spectralis-OCT or different OCT devices and analysing right as well left eyes are recommended, e.g. in the Gutenberg Health Study [40], the LIFE-Adult-Study [41, 42] or UK Biobank [43]. These studies also have data on neurological and cognitive phenotypes and, therefore, could analyse the influence of such disorders on CRV and mortality risk.

Fourth, non-significant results for the associations of CRV with CVD and premature mortality should be interpreted with caution because these two groups had the lowest number of events and, therefore, less statistical power.

The strengths of our study are (i) the analysis in the old-aged population with a median mortality follow-up of 5.9 years and (ii) the stratification for cardiovascular and non-cardiovascular as well as premature and late mortality.

## Electronic supplementary material

Below is the link to the electronic supplementary material.

Supplementary Material 1

## Data Availability

The individual data generated and analysed during the current study are not publicly available due to data privacy of study participants. Summary statistics are available from the corresponding author on request.
