# Supplementary material for: Central retinal volume derived from optical coherence tomography as a potential predictor of mortality in the old-aged population– results from the German AugUR study
Source: Graefes Arch Clin Exp Ophthalmol. 2025 Aug 5;263(10):2737–46. doi: 10.1007/s00417-025-06924-4 (PMC12583423; doi:10.1007/s00417-025-06924-4)
Supplement: Supplementary file 1 — Supplementary Material 1 [file 417_2025_6924_MOESM1_ESM.docx]

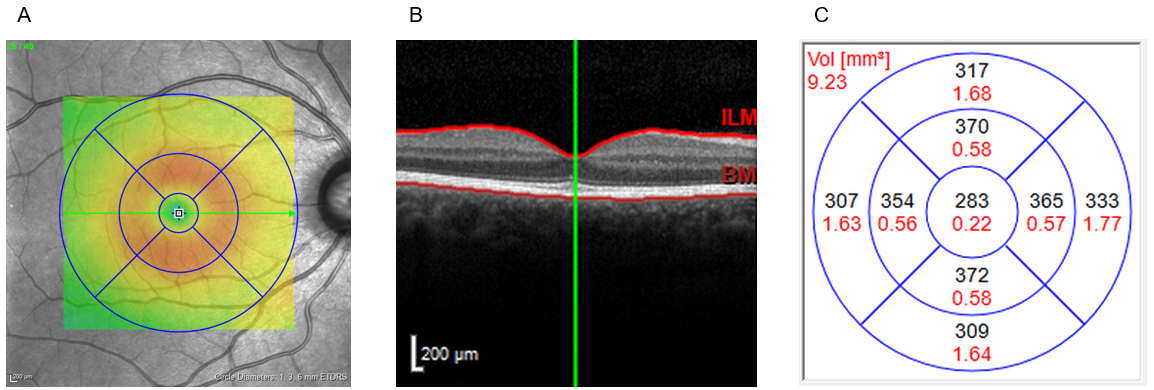


**Supplementary Fig 1** OCT image of the central retina in Heidelberg Eye Explorer and volume calculation. (A) Position of the ETDRS segments on the retina. (B) The central retinal volume (CRV) is calculated from the Bruch’s membrane (BM) to the inner limiting membrane (ILM). (C) Automatic calculation of thickness (in µm, black) and volume across all nine segments (in mm³, red). Example data from the AugUR study with a CRV of 9.23 mm³





**Supplementary Fig 2** Distribution of central retinal volume (CRV) on the mm³ scale of the right eye in different quality control steps. (A) Distribution before any corrections (n=2,192) with mean ± standard deviation (SD) 8.39 ± 0.71 and a range of 12.21 mm³. (B) Distribution after removing OCT sans with ART <25 and quality value <15 (n=2,187) with mean ± SD = 8.39 ± 0.69 and a range of 12.21 mm³. (C) Distribution after removing OCT sans with bad segmentation (n=2,182) with mean ± SD = 8.41 ± 0.62 and a range of 8.15 mm³. (D) Distribution after restriction to ± 4 SD (n=2,166) with a range of 4.88 mm³.


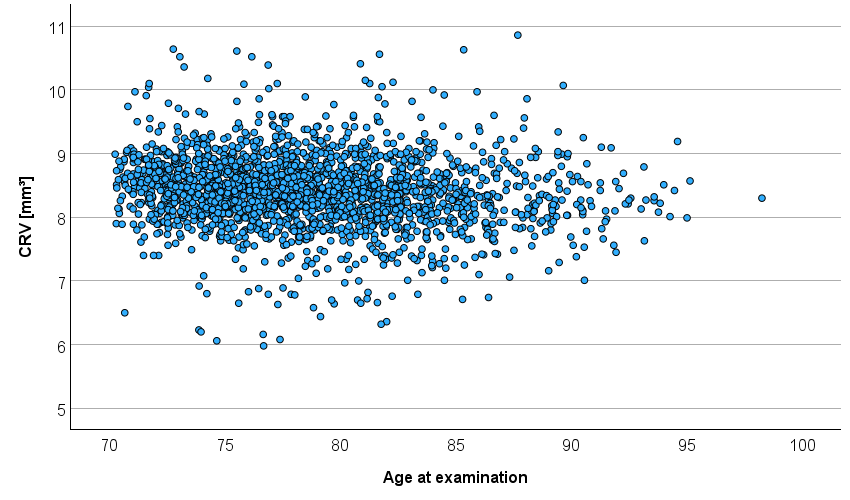


**Supplementary Fig 3** Distribution of central retinal volume (CRV) over age from 2,166 right eyes. Correlation between age and CRV was r = -0.12 (CRV decrease with age)


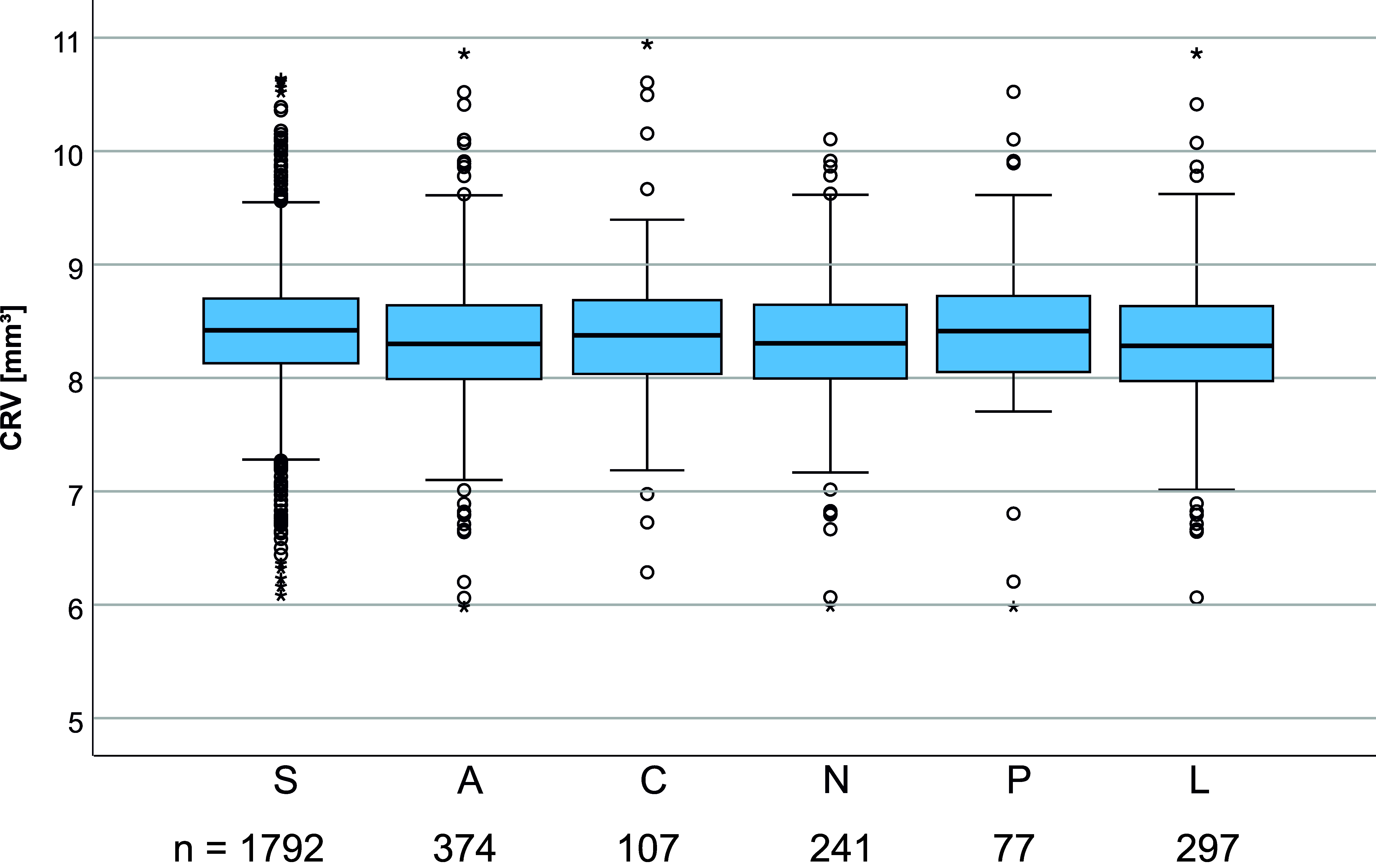


**Supplementary Fig 4** Box plots of central retinal volume (CRV) for mortality groups (S, survived; A, all-cause mortality; C, CVD mortality; N, non-CVD mortality; P, premature mortality; L, late-life mortality). Below the graph, the number (n) of participants per group is shown


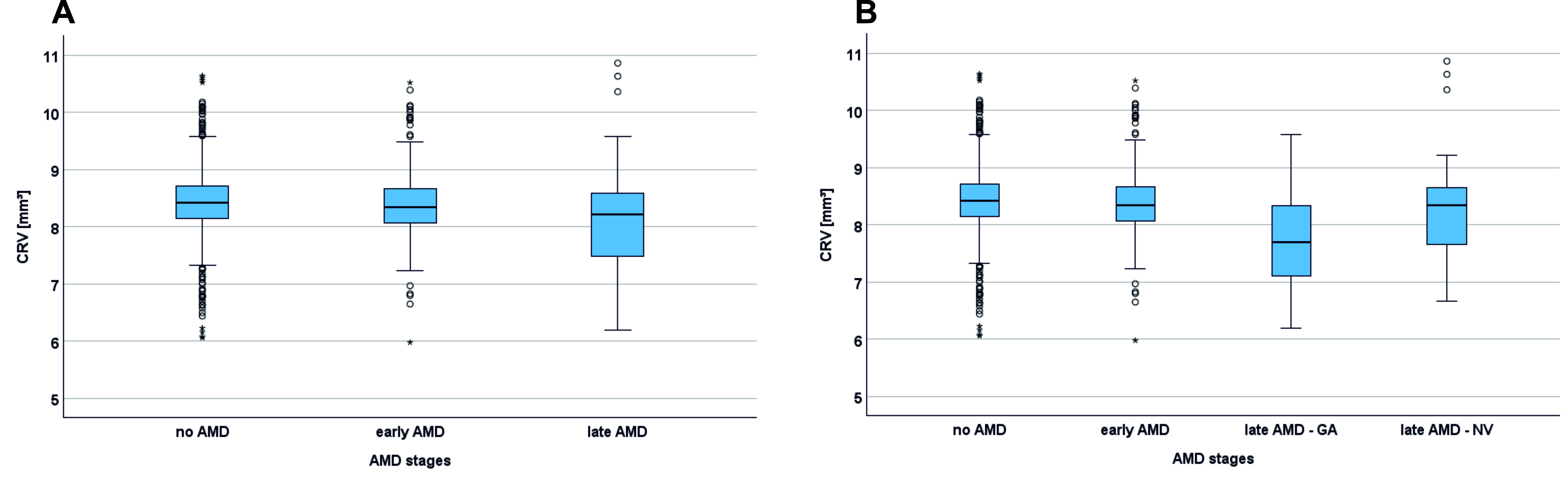


**Supplementary Fig 5** Distribution of central retinal volume (CRV) in different AMD stages. (A) Box plots with comparison of CRV in participants’ right eyes with no (n=1,511), early (n=390) and late AMD (n=99). (B) Box plots with comparison of CRV in participants’ right eyes with no, early and late AMD separated in geographic atrophy (GA, n=23) and neovascularisation (NV, n=73). Note: Lower CRV in late AMD values are mainly due to GA in this study.


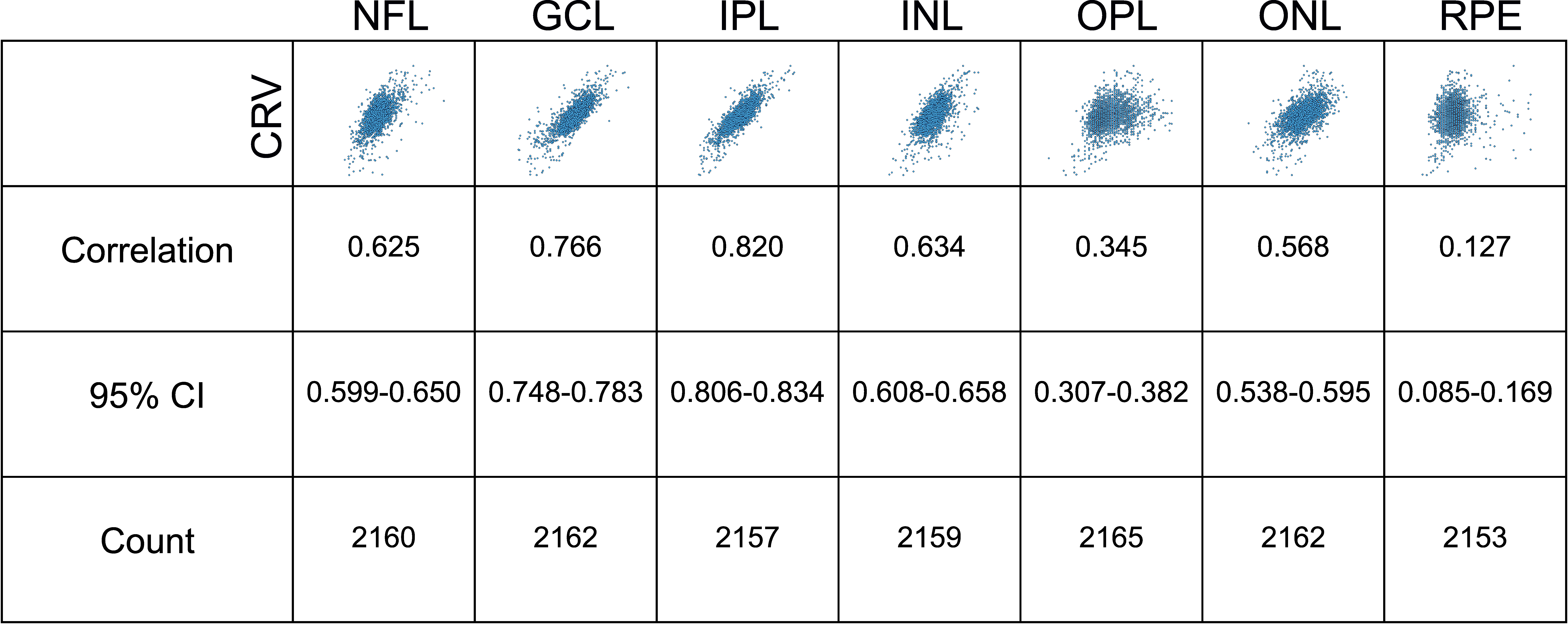


**Supplementary Fig 6** Correlations of central retinal volume (CRV) with seven distinct retinal layer volumes from Heidelberg Spectralis SD-OCT output (NFL, nerve fibre layer; GCL, ganglion cell layer; IPL, inner plexiform layer; INL, inner nuclear layer; OPL, outer plexiform layer; ONL, outer nuclear layer; RPE, retinal pigment epithelium). Depicted are the scatter plots. Person’s correlation coefficients are given with 95% confidence intervals (CI). Count describes the number of data points after quality control based on 2,166 CRV measurements. Note: retinal layers were not manually curated
